# The Uptake of Soluble and Particulate Antigens by Epithelial Cells in the Mouse Small Intestine

**DOI:** 10.1371/journal.pone.0086656

**Published:** 2014-01-27

**Authors:** Savannah E. Howe, Duane J. Lickteig, Kyle N. Plunkett, Jan S. Ryerse, Vjollca Konjufca

**Affiliations:** 1 Department of Microbiology, Southern Illinois University, Carbondale, Illinois, United States of America; 2 Department of Chemistry, Southern Illinois University, Carbondale, Illinois, United States of America; 3 Department of Pathology, Saint Louis University School of Medicine, Saint Louis, Missouri, United States of America; Charité-University Medicine Berlin, Germany

## Abstract

Intestinal epithelial cells (IECs) overlying the villi play a prominent role in absorption of digested nutrients and establish a barrier that separates the internal milieu from potentially harmful microbial antigens. Several mechanisms by which antigens of dietary and microbial origin enter the body have been identified; however whether IECs play a role in antigen uptake is not known. Using in vivo imaging of the mouse small intestine, we investigated whether epithelial cells (enterocytes) play an active role in the uptake (sampling) of lumen antigens. We found that small molecular weight antigens such as chicken ovalbumin, dextran, and bacterial LPS enter the lamina propria, the loose connective tissue which lies beneath the epithelium via goblet cell associated passageways. However, epithelial cells overlying the villi can internalize particulate antigens such as bacterial cell debris and inert nanoparticles (NPs), which are then found co-localizing with the CD11c+ dendritic cells in the lamina propria. The extent of NP uptake by IECs depends on their size: 20–40 nm NPs are taken up readily, while NPs larger than 100 nm are taken up mainly by the epithelial cells overlying Peyer's patches. Blocking NPs with small proteins or conjugating them with ovalbumin does not inhibit their uptake. However, the uptake of 40 nm NPs can be inhibited when they are administered with an endocytosis inhibitor (chlorpromazine). Delineating the mechanisms of antigen uptake in the gut is essential for understanding how tolerance and immunity to lumen antigens are generated, and for the development of mucosal vaccines and therapies.

## Introduction

The mucosa of the gastro-intestinal tract is continuously exposed to dietary and microbial antigens. As an interface between the outside environment (lumen) and the inner body, gut-associated lymphoid tissue (GALT) maintains a delicate balance of inducing immunity against pathogens and tolerance to the antigens originating from the diet and intestinal microflora [1], [2], [3]. Among other factors, the route of antigen uptake and the nature of the antigen dictate the ensuing immune responses in the deeper lymphoid tissues. Lymphoid tissues of the small intestine (SI), such as Peyer's patches, contain M cells that take up large antigens (bacteria, particles, etc.) and deliver them to the underlying immune cells to initiate immune responses [4]. Dendritic cells (DCs) of the SI lamina propria (LP), namely CD11c+ [5], CD103+ [6] and CX3CR1+ [7] DCs extend their dendrites between the IECs and sample lumen antigens. Goblet cell-associated passageways (GAPs) allow the entry of soluble protein antigens, but not inert particles (0.02–2 µm) into the LP [8]. Epithelial cells overlying the GALT and the LP represent a physical barrier that separates the body from the lumen and are also the first point of contact between the host immune system and lumen antigens. Conventional IECs (enterocytes) absorb digested, small molecular weight dietary antigens via the transcellular pathway [9], [10], [11] and allow small molecules and inert experimental probes (5–10 Å) to access the LP via the tight junctions (paracellular pathway) [9], [12], [13]. IECs differ from M cells in that they contain closely packed microvilli [14] and express 400–500 nm-thick mucin-like glycoproteins that form a layer covering the tips of the microvilli; whereas M cells lack microvilli, do not secrete mucus and generally lack the thick glycocalyx layer [15], [16], [17]. The mucus layer traps microorganisms and other large inert antigens, preventing their direct contact with the IEC membranes [2], [18], [19] and access to inter-microvillar endocytic domains [15]. However, smaller pathogens such as norovirus (20–30 nm) and human papilloma virus (∼55 nm) can readily diffuse through cervical mucus [20] which is similar in physical properties to the mucus covering the IECs [21]. Whether IECs play an active role in the uptake and sampling of small particulate lumen antigens such as viruses and bacterial cell debris in vivo is not known. Mathiowitz and coworkers showed that polymer particles 40–120 nm in size, engineered to display strong adhesive interactions with mucus and cell membranes are taken up by IECs and facilitate the transport of conjugated substances into the LP [22]. In contrast, larger size polystyrene and poly(lactic acid) particles are taken up exclusively by M cells associated with Peyer's patches [23], [24], [25]. Here we used fluorescently labeled antigens and polystyrene NPs to examine their in vivo uptake by confocal and immunofluorescence microscopy (IFM). We report that routes of antigen uptake depend on the nature of the antigen. Soluble, small molecular weight antigens enter the LP via GAPs, while 20 and 40 nm NPs cross the mucus layer and are internalized by the IECs of the villi. Internalized NPs are then found in the underlying CD11c+ LP DCs, blood, and lymphatic ducts of the villi through which they reach the mesenteric lymph nodes (MLNs).

## Materials and Methods

### Ethics statement

All animal experiments were performed in accordance with NIH guidelines, the Animal Welfare Act, and US federal law. Animals were housed in centralized AAALAC-accredited research animal facilities, staffed with trained husbandry, technical, and veterinary personnel. All experiments were conducted in accordance with protocols approved by the Southern Illinois University Institutional Animal Care and Use Committee and Saint Louis University Animal Studies Committee.

### Animals, model antigens, reagents and antibodies used

Six to ten week-old C57BL/6 mice (Jackson laboratories) were used for the studies. Carboxylate-modified fluorescent polystyrene NPs, ranging in size from 20 nm to 2 µm (Invitrogen), and *E.coli* BioParticles® (Invitrogen) were used as model particulate antigens. Chicken Ova (45 kDa, Sigma), Ova-fluorescein conjugate (Invitrogen), dextran-fluorescein, lysine-fixable dextran-biotin (40 kDa, Invitrogen), and LPS-Alexa Fluor® 488 (3 kDa, Invitrogen) were used as model soluble antigens. Biotinylated rabbit anti-Ova antibodies (Thermo Scientific) and streptavidin-FITC (Biolegend) were used to detect Ova and Ova conjugated to NPs (NP-Ova). Anti-CD11c (eBioscience), Cy-18 (Biolegend) and Lyve-1 (eBioscience) antibodies were used to label LP DCs, goblet cells, and lymphatic ducts respectively. A combination of monoclonal mouse anti-E-cadherin (BD Biosciences) primary antibody and goat anti-mouse-FITC (BD Biosciences) secondary antibody was used to label the IECs. All antibodies were used at a 1∶100 dilution in appropriate blocking buffer. To highlight the tissue architecture in cryosections, actin-binding Phalloidin-Alexa 350 (Invitrogen) was used. DAPI (4′,6-Diamidino-2-Phenylindole, Dilactate, Invitrogen) was used for in vivo labeling of the IEC nuclei. Genistein and chlorpromazine (CPZ) (Sigma) were used for in vivo inhibition of NP uptake at 200–1000 µM and 10–100 µg/ml respectively.

### Administration of antigens to the mice

For short-term experiments 50–100 µl of PBS containing ether NPs (1–10%), *E.coli* particles (0.1–1 mg) and/or soluble antigens (dextran-fluorescein (0.5–2 mg), Ova-fluorescein (0.2–2 mg), LPS-Alexa Fluor® 488 (15–20 µg) were administered directly into the SI lumen of anesthetized mice, no further than 10 cm upstream of the ileo-cecal junction. For this, mice were fasted for 2 hours then anesthetized with isoflurane delivered in a stream of oxygen. The SI was exposed via a small incision in the abdominal wall and antigens were injected using a 30 g needle. During anesthesia animals were placed on a warming pad and covered with a cloth to maintain a stable body temperature. In some experiments antigens were administered in a 200 µl volume of PBS via a gastric gavage using a round-tip needle. Before gastric gavage, mice were fasted for 2–3 hours.

### In vivo imaging of the SI

At pre-determined times after antigen administration to the SI (or via a gastric gavage), a small incision was made in the wall of the SI. The lumen side of the SI was secured under a cover glass with a thin film of Vetbond (Ted Pella). To inhibit intestinal peristalsis mice were injected sub-cutaneously with 200 µl of PBS containing 1 mg/ml scopolamine 15–30 minutes before imaging. The mouse was then placed in a custom-made restraint device on the microscope stage of an Olympus FV1000 MPE scanning confocal microscope and imaged using a 20× 0.95 NA water immersion objective (Olympus). Fluorophores were excited using a Chameleon UltraII laser (Coherent) with excitation wavelengths from 640 to 1080 nm. Ten to 50 sequential Z-stacks were acquired at ∼1–2 µm spacing, rendered into 3D images using Volocity software (Perkin Elmer) and examined for the presence of fluorescent particles in IECs, LP, and the MLNs. In some experiments dextran-fluorescein was injected i.v. in a tail vein 30 min before in vivo imaging to highlight blood and lymphatic vessels in the SI.

### Analysis of particle internalization by immunofluorescent microscopy (IFM)

Parallel experiments were performed for analysis of NP internalization and location within the tissues by IFM. At different times after NP administration (per-orally or in the SI) mice were euthanized and sections of the SI were excised and snap-frozen in Tissue-Tek® O.C.T. freezing compound on dry ice. Tissue cryosections (5–7 µm thick) were fixed in 4% paraformaldehyde (PFA), washed with PBS then incubated with blocking buffer (Thermo Scientific) for 10–15 minutes. After blocking, sections were stained with antibodies or phalloidin and imaged with a Leica DM4000B fluorescent microscope. Images were analyzed with Volocity software. For TEM imaging sections of the SI were prepared as described previously [26].

### Quantification of NPs accumulated in the MLNs

In these experiments 40 nm NPs were administered to the lumen of the SI with or without CPZ. Forty minutes after NP administration mice were euthanized, MLNs were excised, and draining lymphatic ducts and the adipose tissue surrounding the MLNs were removed under a dissecting microscope. Fluorescence intensity of the isolated MLNs was measured with a Shimadzu RF-5301 PC spectrofluorophotometer. Diluted 40 nm NPs in PBS were used to set a calibration curve for the instrument.

### Quantification of NPs accumulated in the MLNs using Volocity

Experiments were conducted as described in the above paragraph. Excised MLNs were snap-frozen in OCT on dry ice then 7 µm sections of the MLNs were imaged with a Leica DM4000B fluorescent microscope at 630× magnification. Acquired images from all MLN compartments were analyzed with Volocity software. Care was taken to quantify pixels specific for fluorescent NPs only within the MLN tissue. The number of pixels specific for fluorescent NPs was expressed as the percentage of pixels per image (MLN tissue surface area). Data acquired from 10 representative images per mouse taken at 630× magnification from MLN compartments (high or low NP concentration) were used for statistical analysis.

### Conjugation of particles with ovalbumin

n a 2 mL eppendorf tube 200 µl of NPs (2 wt % solid solution) were mixed with 800 µL of 100 mM PBS solution and 8.0 mg of Ova (Sigma). The mixture was incubated at room temperature for 15 minutes and then 8.0 mg of 1-ethyl-3-(3-dimethylaminopropyl) carbodiimide-hydrochloride (EDAC-HCl) was added. The reaction mixture was incubated for 2 hours at room temperature with regular agitation. The resulting particle dispersion was then dialyzed using a Float-A-Lyzer® membrane (100 kDa cutoff) for 3 days against 100 mM PBS (pH 7.4) that was changed daily. The resulting protein-conjugated NPs were diluted in PBS and stored at 4°C until used.

### Analysis of NPs conjugated to OVA immobilized on a nylon membrane

To confirm that the NPs were conjugated to Ova, NPs, Ova, or Ova-conjugated NPs (NP-Ova) were spotted on a 0.2 µm nylon membrane. The membrane was washed with Tris-buffered solution containing 0.1% Tween 20 (Sigma) (TTBS), blocked with TTBS containing 5% skim milk (Difco) for 1 hour then incubated with biotinylated rabbit anti-Ova antibodies (Thermo Scientific) for 1 hour. After 3 washes the membrane was incubated with streptavidin-FITC (Biolegend) for 1 hour, washed, dried then imaged with a Leica DM4000B fluorescent microscope using a 2.5× objective.

### Isolation of epithelial cells from the SI

Sections of the SI were flushed with cold PBS and the Peyer's patches were excised and discarded. The SI was cut longitudinally, then into 2 cm sections and placed in 250 ml Erlenmeyer flasks containing 40 ml of pre-warmed (37°C) HBSS (HyClone) supplemented with 5 mM EDTA. Flasks were incubated for 15 minutes on a shaking rotator at 37°C. Supernatant containing IECs was passed through a 70 µm cell strainer (BD Falcon) and IECs were pelleted by centrifugation. Pelleted IECs were washed 3 times with RPMI-10 (HyClone) supplemented with penicillin (100 u/ml) and streptomycin (100 µg/ml). To confirm that isolated cells were IECs they were fixed in 4% PFA, washed with PBS then incubated with mouse anti-E-cadherin antibodies for 1 hour. IECs were washed in PBS then incubated with FITC-conjugated goat anti-mouse antibodies for 1 hour. Isolated IECs were placed on a glass slide and imaged with a fluorescent microscope. A combination of the same antibodies was used to confirm the expression of E-cadherin in isolated IECs by western blot analysis. As a negative control for E-cadherin expression, isolated spleen lymphocytes were used.

### Statistical analysis

Each experiment was repeated at least 3 times. Data were analyzed using procedures of SAS software. Group means were separated by using Student's t-test and were considered significantly different at P<0.05. Data are expressed as mean ± standard deviation (SD) of the mean.

## Results

### Entry of small molecular weight antigens into the LP via GAPs in vivo

**Figure 1:**
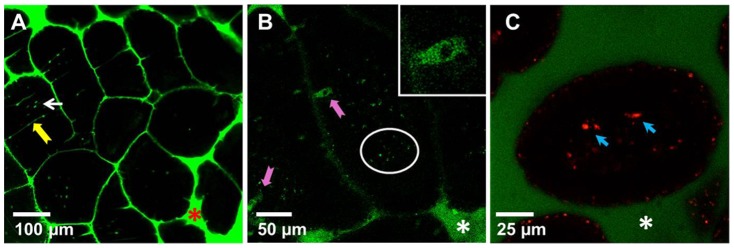
The uptake of soluble and particulate antigens by epithelial cells of the mouse small intestine in vivo. Intestines of anaesthetized mice were injected intraluminally with (A) dextran-fluorescein (green), (B) LPS-Alexa 488 (green), (C) dextran-fluorescein (green) and *E.coli* cell debris (red). Thirty minutes to 1 hour later luminal surfaces of the SI were imaged with intravital confocal microscopy. Images of optical sections revealed dextran (A, (arrows)), LPS (B) in the SI lumen and entering the LP (circled) via GAPs (pink arrows, inset). (C) *E.coli* cell debris accumulates in the LP of the villi (blue arrows); Asterisks in all panels denote the lumen of the SI. Each image is a representative of at least 3 experiments.

In vivo imaging allowed us to examine the uptake and distribution of antigens in the SI in a living mouse with an intact epithelial barrier and uninterrupted blood and lymphatic flow. The routes of antigen administration and antigen uptake are summarized in Table 1. As expected, the small molecular weight dextran (40 kDa) and Ova (45 kDa) entered the GAPs and the LP when injected into the intestinal lumen (Figure 1A (yellow arrow)) [8]. *Salmonella* LPS (3 kDa) also entered via GAPs (Figure 1B (pink arrows, inset)) and accumulated in the LP of the villi (Figure 1B (circled)). We then sought to examine whether larger particulate bacterial antigens enter the LP via GAPs by using fixed, fluorescent *E.coli* as a model antigen. Cell debris, generated by sonicating *E. coli* (but not intact bacteria) entered the LP of the villi (Figure 1C (blue arrows)). We did not observe any significant amount of *E.coli* cell debris entering the LP via GAPs, indicating that the antigen size was important for their entry into the LP via GAPs.

**Table 1:** Routes of uptake (entry) of soluble and particulate antigens in the small intestine (SI) of mice.

		Routes of antigen uptake in the SI		
	Mode of administration	GAPs	IECs	Peyer's patches
**Ova**	PO, IL	+	-	+
**Dextran**	PO, IL	+	-	+
**LPS**	IL	+	-	N/E
**NPs<40 nm**	PO, IL	N/E	+	+
**NPs>100 nm**	PO, IL	-	-	+

Abbreviations: PO: Per-oral; IL: Intraluminal (injected in the lumen of the SI); N/E: Not evaluated. GAPs (Goblet Cell Associated Passageways); IECs (Intestinal Epithelial Cells).

### Differential uptake of soluble and particulate antigens by IECs and GAPs

**Figure 2:**
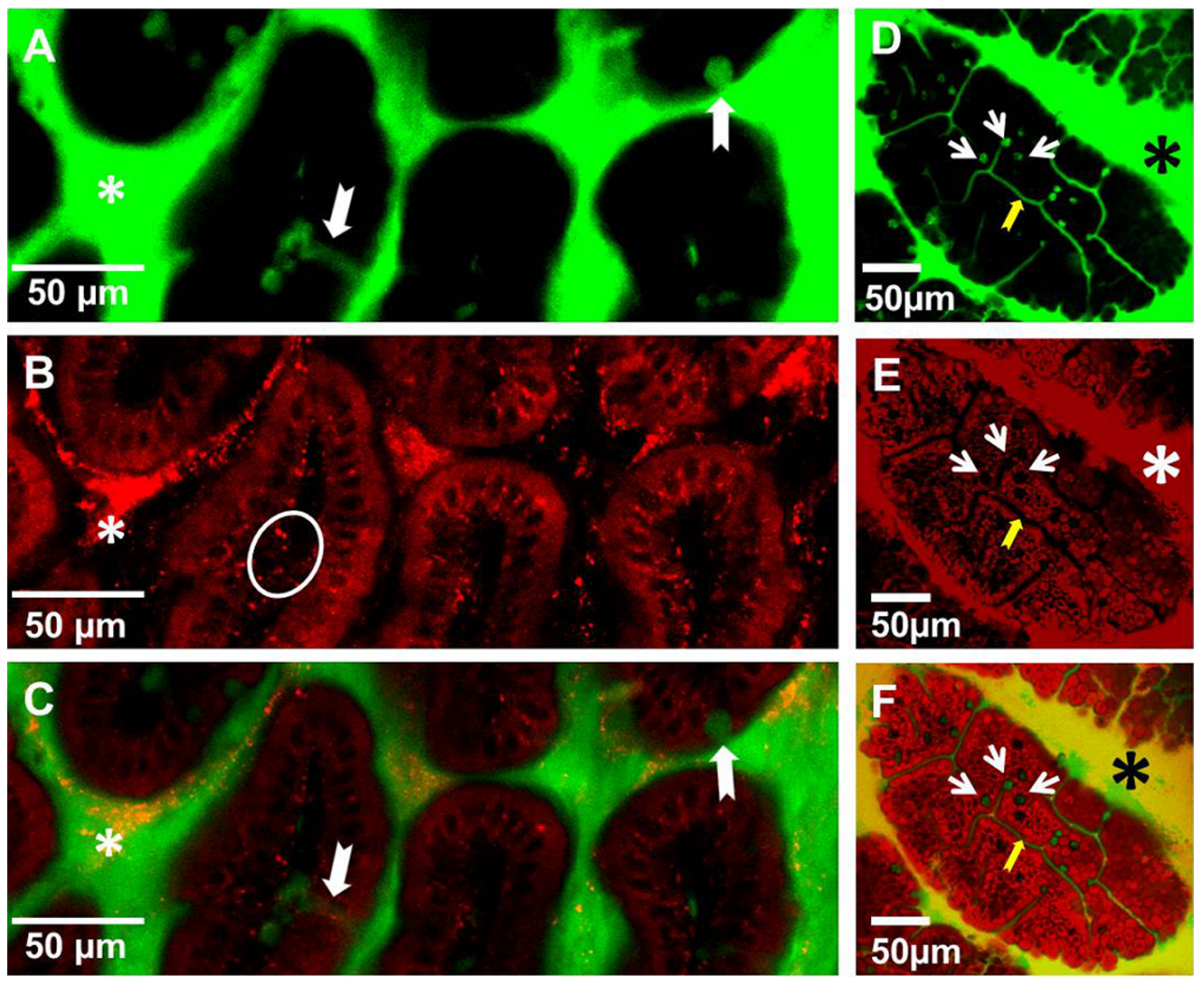
Differential uptake of per-orally administered soluble and particulate antigens in the villi of the SI in vivo. Mice were co-administered (A–C) dextran-fluorescein (green) and 20 nm NPs (red) or (D-F) Ova-fluorescein (green) and 20 nm NPs (red) per-orally and 30–40 minutes later the SI was imaged from the lumen side with a confocal microscope. (A–C) A 1.5 µm Z-stack image that depicts a cross-section of the villi is shown. (A) Lumen dextran-fluorescein (green, asterisk) enters the LP of the villi via GAPs (white arrows); (B) NPs are observed on the basolateral side of IECs and in the LP (circle). (C) Overlap of images A and B. (D–F) Conduit-like structures (yellow arrows) and GAPs (white arrows) are highlighted by Ova-fluorescein (green). (E, F) IECs (red) are highlighted by NPs but not GAPs which appear as black holes (E) (white arrows); (F) Overlap of images D and E. Asterisks in all panels denote the lumen of the SI. Images are representative of at least 3 experiments.

**Figure 3:**
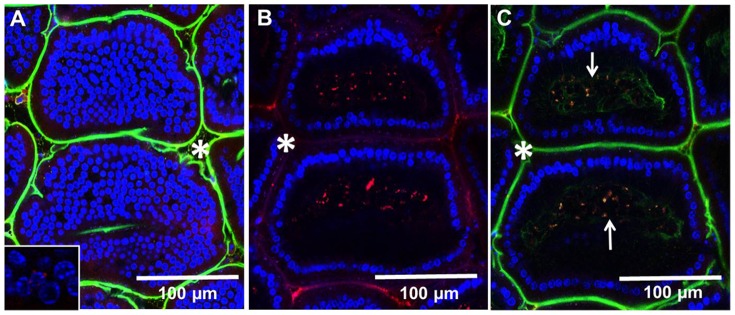
Localization of 40 nm NPs in the circulation of the villi 30 minutes after administration in the SI lumen in vivo. To highlight the vasculature and lymph ducts of the villi, dextran (green) was injected in the tail vein while NPs (red) and DAPI (blue) were injected into the lumen of the SI. Thirty minutes later the SI was imaged with a confocal microscope and sequential Z-stacks were acquired. (A) A Z-stack image taken at the tip of the villi: nuclei of the IECs above the LP stained with DAPI (blue) and lumen (asterisks) stained with dextran (green) that has leaked from circulation. Inset: a magnified image showing the proximity of NPs to the IEC nuclei. (B) A Z-stack of the same villi taken at 70 µm depth showing NPs (red) that have accumulated in the LP and IEC nuclei stained with DAPI (blue) surrounding the LP. (C) Three color Z-stack shown in B in which NPs are seen co-localizing with vasculature of the villi (arrows). The images are representatives of at least 3 experiments.

**Figure 4:**
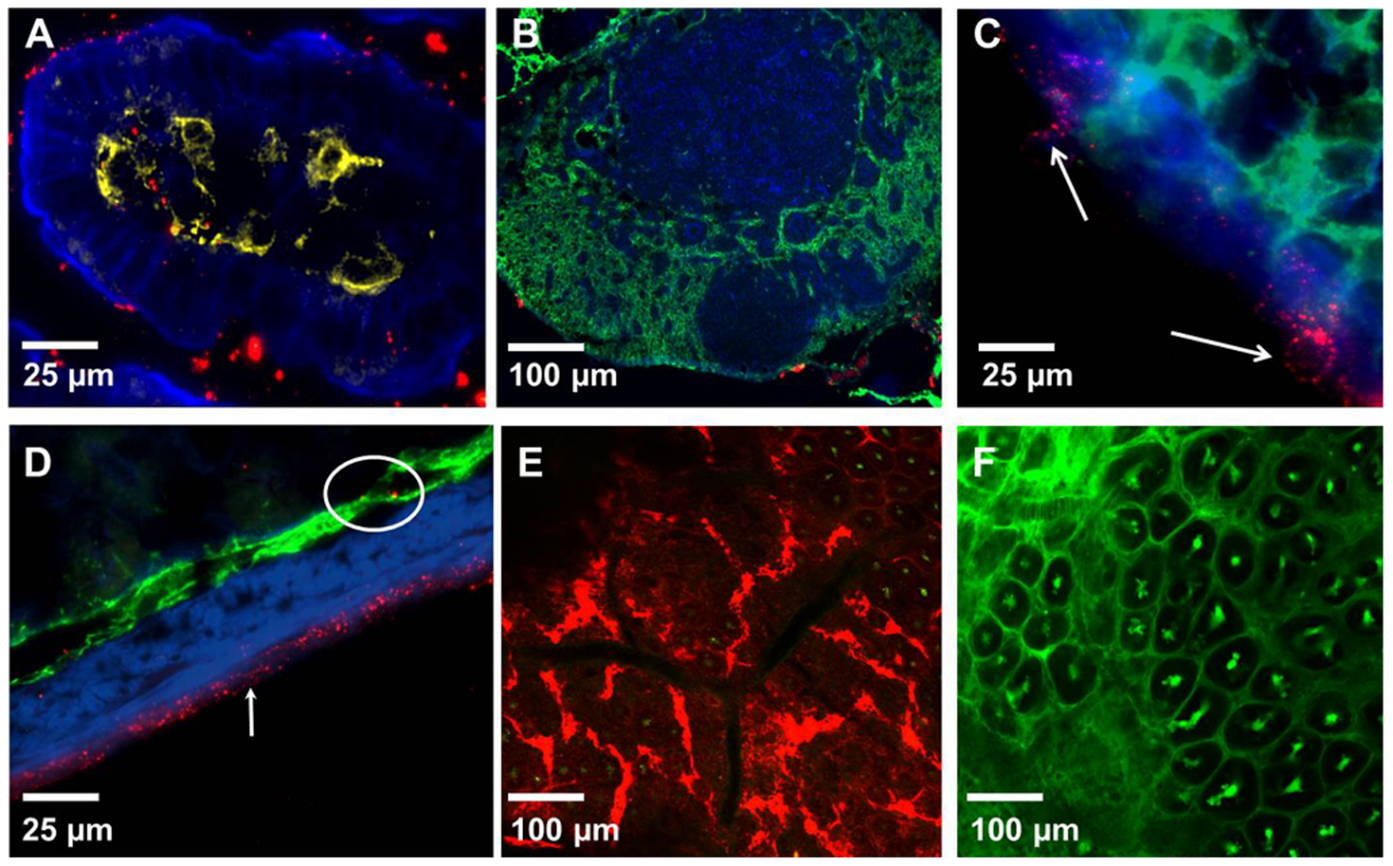
Distribution of NPs in the SI and MLNs of mice 30–40 minutes after administration in the SI. (A) A three color IFM image of a villus showing co-localization of NPs (red) with CD11c+ DCs (yellow) in the LP. Actin highlighted by phalloidin-Alexa 350 (blue). (B) A three color IFM image of a MLN 40 minutes after NP administration into the SI. NPs (red) seen in the capsule of the MLN. The lymphatics of MLN stained with Lyve-1 antibodies (green). Actin is highlighted by phalloidin-Alexa 350 (blue). (C) A higher resolution three color image of MLN capsule taken at 630×. Some NPs (red) appear to be cell-bound (arrows). (D) A three color IFM image of an intestinal section. NPs (red) colocalize with the lymphatics (Lyve-1 staining, green, circled) in the submucosa and highlight the serosa (white arrow). Actin is highlighted by phalloidin-Alexa 350 (blue). (E) A confocal image of the SI serosa taken in vivo 40 minutes after NP (red) and dextran-fluorescein (green) administration. (F) A confocal image of the SI serosa taken in vivo 40 minutes after administration of dextran-fluorescein (green, control). Each image is a representative of at least 3 experiments.

**Figure 5:**
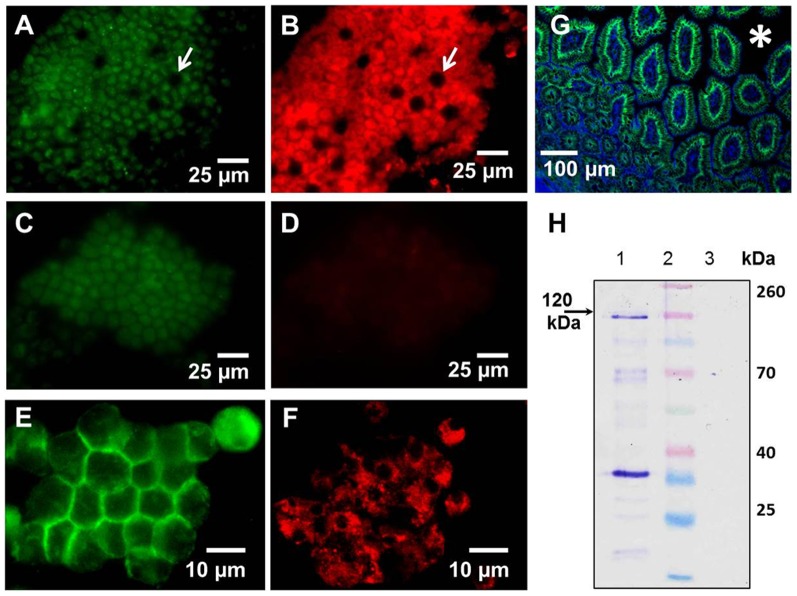
The presence of NPs in the IECs isolated from the mouse SI. 40(red) were injected into the lumen of the SI and 30 minutes later the SI was excised, Peyer's patches were removed (discarded), and IECs were isolated from the SI sections. (A–D) Isolated IECs from mice that were administered NPs (A, B) or PBS (C, D) were fixed then placed on a glass slide and imaged with a fluorescent microscope at 630× magnification. (A, B) A patch of IECs isolated from NP-treated mouse imaged in the green channel (autofluorescence) (A) and the red channel (red: NPs) (B). Characteristic GAPs that are not highlighted by NPs appear as black holes in isolated IEC patches (white arrows), while IECs exhibit strong red fluorescence due to the presence of NPs (similar to images taken in vivo). (C, D) No red fluorescence was detected in IEC patches isolated from a control mouse. (E) Expression of E-cadherin (green) in isolated IECs imaged with a fluorescence microscope. (F) A confocal image of IECs isolated from NP-treated mouse showing strong red fluorescence in IEC cytoplasm. (G) Distribution of E-cadherin (green) in a section of SI. Actin staining with phalloidin-Alexa 350 (blue) highlights the tissue architecture. (H) Western blot analysis of E-cadherin (120 kDa) expression in isolated IECs (Lane 1) or spleen lymphocytes (Control, Lane 3). Lane 2: Spectra™ multicolor protein ladder. Each image is a representative of at least 3 experiments.

To examine how the particle size might affect their uptake, in further studies we used negatively charged fluorescent polystyrene particles ranging in size from 20 nm to 2 µm. These particles are available in a variety of sizes, do not bind to cells, and are impregnated with fluorophores, which makes them suitable for in vivo imaging and IFM analysis. To examine whether NPs enter the LP via GAPs we co-administered NPs with either dextran or Ova per-orally. This approach enabled us to simultaneously examine the entry of NPs and fluorescent soluble antigens into the LP. Within the time examined (30 minutes to 1 hour after administration) IECs did not internalize any appreciable amount of Ova or dextran. Within 30–40 minutes of per-oral administration dextran reached the LP of the villi via GAPs (Figure 2 A, C (white arrows)), while NPs appeared to be internalized by IECs and were found on their basolateral side in the LP (Figure 2 B (circled), C, Movie S1 and S2). No red fluorescence was observed in IECs (Figure 1A) of control mice to which no NPs were administered, although the imaging was conducted under identical settings. Ova reached the SI within 30 minutes of per-oral administration and like dextran entered the GAPs, giving them a green appearance (Figure 2 D, F (white arrows)), while NPs were internalized by IECs, giving them a red appearance (Figure 2 E, F). GAPs do not appear to take up any appreciable amount of NPs and appear as “black holes” in the red channel (Figure 2 E (white arrows)). The entry of Ova and dextran into the GAPs appeared to be guided by a network of conduit-like structures present on the surface of the villi (Figure 2 D–F (yellow arrows), Movie S3 and S4). Conduit-like structures were also observed in the Peyer's patches (Figure S1 A, B). These structures become apparent when highlighted by fluorescent antigens. Using TEM we observed that NPs were lodged between the microvilli (Figure S2 A, B) of the IECs, and did not appear not to be uniform in size (Figure S2 B). This could be because the specified sizes of NPs available commercially represent their nominal bead diameters. However, due to their very small size, 20 and 40 nm NPs are less uniform in size when manufactured, with a higher coefficient of variation (up to 20% (± nominal diameter)), Invitrogen). Because of batch variation during manufacturing it is possible that occasional larger particle is found in the mix of NPs. Within the epithelial cells NPs were found in close proximity to the DAPI-stained nuclei of IECs (Figure 3 A (inset), Figure S4 A) and co-localizing with the vessels of the villi (Figure 3 B, C (white arrows), Movie S5). In the LP of the villi NPs co-localized with CD11c+ DCs (Figure 4 A) and within 30-40 minutes of administration into the SI, NPs were observed in the MLNs (Figure 4 B, C), serosa of the SI visualized in tissue cryosections (Figure 4 D) and in the SI serosa in vivo (Figure 4 E). No red fluorescence was observed in SI serosa of control mice to which only dextran (green) was administered (Figure 4 F). We then isolated the IECs from Peyer's patch-free sections of the SI at 30 or 40 minutes after NP administration and repeatedly observed strong red fluorescence in the isolated IECs of NP-administered mice (Figure 5 B). To show a presence of cells in IEC preparations we upregulated the signal in the green channel (Figure 5 A, C). Shorter incubation times of SI sections enabled us to peel off intact patches of epithelium with appearance of GAP locations as black holes, resembling their appearance in vivo (Figure 5 A, B vs. Figure 2 D–F (white arrows)). No red fluorescence was observed in IECs isolated from the control mice that were given PBS (Figure 5 C, D), ruling out the possibility that the red fluorescence observed in vivo is due to the autofluorescence of IECs. Confocal imaging of isolated IECs revealed that NPs were found within their cytoplasm (Figure 5 F). Confocal images of IECs isolated from controls did not have any signal in the red channel, thus appeared completely dark and were not shown in figure. Microscopic examination revealed that the great majority of isolated IECs (about 90%) were positive for E-cadherin (Figure 5 E), a 120 kDa transmembrane glycoprotein that is localized in adherens junctions of epithelial cells (Figure 5 G). The expression of E-cadherin in isolated IECs was also confirmed by western blot analysis (Figure 5 H). As a negative control for E-cadherin expression we used lymphocytes isolated from the spleen (Figure 5 H).

### Internalization of NPs can be inhibited by an endocytosis inhibitor

**Figure 6:**
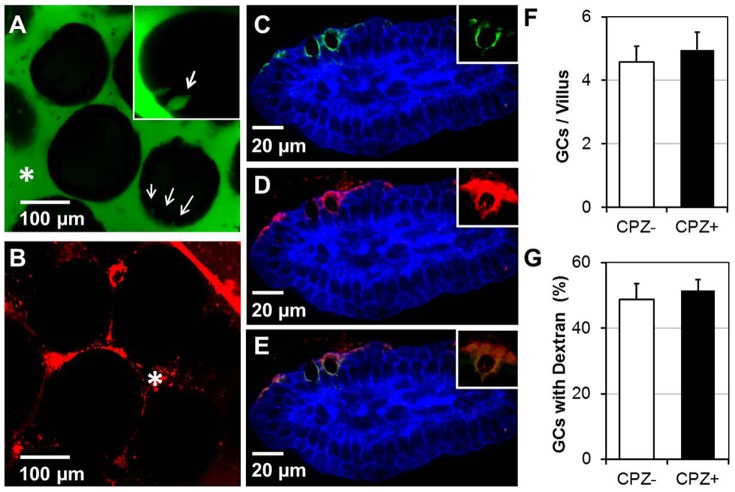
Administration of CPZ inhibits the uptake of 40 nm NPs but does not affect the uptake of dextran via GAPs. (A) Green channel of a confocal image of SI villi taken in vivo showing the entry of dextran (green) into the LP via GAPs (arrows, inset) in CPZ-treated mouse SI. (B) The internalization of 40 nm NPs (red channel) is inhibited by CPZ, thus red fluorescence was detected only in the lumen of the SI (asterisk). (C–E) A representative IFM image of the villi from tissue sections of mice administered CPZ and lysine-fixable dextran (red). Goblet cells (GAPs) were stained with cytokeratin 18 (Cy-18) antibody (green). (C) Two color image showing actin staining (blue) and goblet cell staining (green). (D) A two color image showing the entry of dextran (red) via GAPs. (E) Overlap of image C and D showing co-localization of red dextran with Cy-18 positive GAPs (green). (F) Administration of CPZ does not alter the number of Cy-18+ cells in the villi. There were no differences in Cy-18+ cells present in the villi between CPZ-treated and control mice (p<0.05). (G) Administration of CPZ did not alter the entry of dextran into the LP via GAPs. There were no differences in the number of Cy-18+ GAPs co-localizing with dextran between CPZ-treated and control mice (p<0.05). Group means were separated using Student's t-test and were considered significantly different at P<0.05. Data (bars) are expressed as mean ± SD of the mean. In total over 200 villi and over 600 GAPs were counted per animal and per treatment group (+/− CPZ). For each treatment group 3 mice were used. Data are representative of 3 experiments.

Using TEM we did observe 40 nm NPs lodged between the microvilli of IECs (Figure S2), however due to a lack of contrast within the tissues we had difficulties visualizing NPs when tissues were prepared using standard fixation techniques. This made it difficult to unequivocally identify the endocytic compartments harboring NPs within the IEC cytoplasm. We then administered NPs with either genistein (an inhibitor of caveolae-mediated endocytosis) or CPZ (an inhibitor of clathrin-mediated endocytosis) into the SI and visualized the NP uptake in vivo. In our hands, co-administration of genistein with 20 or 40 nm NPs did not inhibit their uptake by IECs, even when genistein was used at 1 mM, a 5-fold higher concentration than is commonly used for cultured cells (not shown). In contrast to this, co-administration with CPZ did greatly inhibit the uptake of 40 nm NPs by IECs in vivo, even though the amount of CPZ used was 5-fold higher (50 µg/ml) than what is typically used for in vitro studies (Figure 6 A, B). CPZ did not affect the average numbers of GAPs per villi (Figure 6 F) or the dextran entry into the LP via GAPs (Figure 6 C–E, G). We then used 10-fold higher concentration of CPZ, however similarly to previous studies in which lower CPZ concentration was used (Figure 6 A, B), the NP uptake was not inhibited in all areas of the SI examined in vivo. This finding could be due to the fact that NPs were administered in a small volume of PBS (50–100 µl) and that the in vivo absorption of CPZ in some areas may be more extensive than in others.

### Quantification of in vivo NP uptake with and without CPZ inhibition

**Figure 7:**
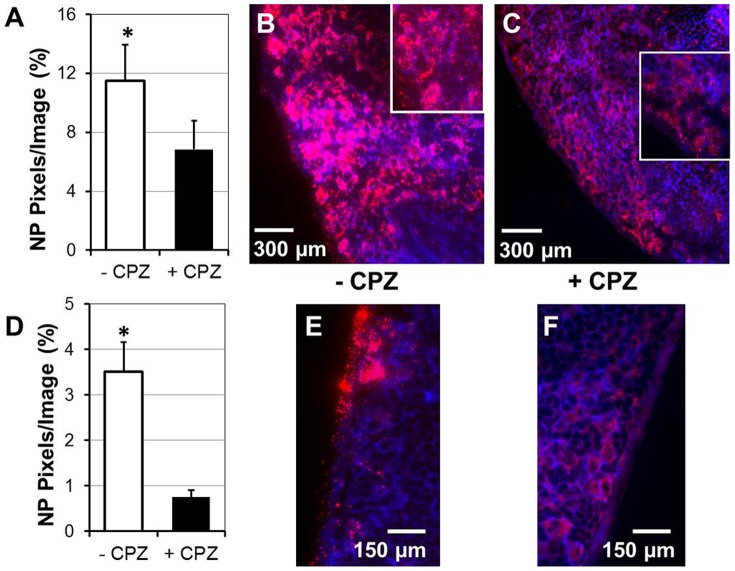
Inhibition of 40 nm NP uptake by CPZ leads to decreased concentration of NPs in the MLNs. NPs with or without CPZ were injected in the lumen of SI and 40 minutes later MLNs were snap-frozen. Tissue sections of MLNs from CPZ-treated and control mice were stained with phalloidin-Alexa 350 and imaged at 630×. The amount of NPs in MLN sections of CPZ-treated and control mice was quantified using Volocity software. Regions of MLNs with highest NP concentration from control and CPZ-treated mice (A–C) were analyzed separately from regions of MLN capsules from control and CPZ-treated mice (D–F). (A) The amount of NPs (red pixels per image) was significantly higher in control mice compared to CPZ-treated mice (p<0.05). (B, C) Stitched images of MLN regions with high NP concentration from tissues of control mice (B) and CPZ-treated mice (C). Eight images taken at 630× were stitched together to show large sections of MLNs. Insets: magnified representative images in which clumps of NPs and individual NPs can be visualized. (D) The amount of NPs (red pixels per image) was significantly higher in capsules of control mice compared to CPZ-treated mice (p<0.05). (E, F) Representative images of NP distribution in MLN capsules of control (E) and CPZ-treated mice (F). Data are representative of 3 experiments (6 mice). Group means were separated using Student's t-test and were considered significantly different at P<0.05. Data are expressed as mean ± SD of the mean.

Parallel experiments were conducted in which 40 nm NPs with or without CPZ were administered to the SI of the anesthetized mice. Forty minutes after NP administration MLNs were either frozen for IFM analysis or were analyzed by spectrofluorophotometry. For IFM analysis, cryosections of entire MLNs were imaged at 630× and the amount of NPs (red pixels) was quanified within the MLN tissue. MLNs of mice that received CPZ had significantly less NPs in regions of high NP concentration (Figure 7 A–C), as well as in MLN capsules (Figure 7 D–F). This finding was confirmed with spectrofluorophotometry (Table 2). For measuring fluorescence intensity in MLNs, we generated a calibration curve using known concentrations of NPs. In all three experiments we observed a lower intensity of fluorescence (U) in MLNs isolated from mice which were administered NPs with CPZ compared to the intensity of fluorescence in MLNs of mice to which only NPs were administered (Table 2).

**Table 2:** Intensity of fluorescence in MLNs of mice 40 minutes after administration of 40

	Intensity of fluorescence (U)		
	**Control**	**- CPZ**	**+ CPZ**
**Exp. 1**	375.8	634.5	461.0
**Exp. 2**	259.5	645.0	260.3
**Exp. 3**	260.0	322.2	254.7
	* **Calibration standards for 40 nm NPs**		
**NPs/mL**	2.2×10^10^	4.4×10^10^	8.9×10^10^
**40 nm NPs (U)**	251.3	390.8	688.4

* Calibration curve for NP concentration was generated using 40 nm NPs diluted in PBS. Concentrations of 40 nm NPs/mL were calculated using the formula provided by the manufacturer. For each treatment 3 mice were used (a total of 9 mice).

### NP size affects their internalization

Regardless of the route of administration (per-oral or injected in the SI), the uptake of smaller particles was more efficient. Peyer's patches internalized a significant amount of NPs (20 nm–100 nm) (Figure S3 A, B), but very few large particles (0.5–2 µm) within the same time frame (not shown). When 20 nm and 40 nm NPs were given per-orally they reached the serosa of the SI in large amounts (Figure S3 C). In contrast, when 40 and 1000 nm NPs were administered, large amount of 40 nm NPs (green), but very few 1000 nm NPs (red) reached the serosa of the SI (Figure S3 D, (arrows)). For the most part, the larger NPs adhered to the mucus and made less direct contact with the epithelial cells overlying the Peyer's patches (not shown) and the villi (Figure S3 E). When 100 nm or 500 nm NPs were administered into the SI they were predominantly found in the lumen and were not internalized by IECs (Figure S4 B–E).

### NPs conjugated to Ova are internalized efficiently and transport Ova to the deeper lymphoid tissues

We then examined whether coating NPs with a protein antigen altered their uptake. For this, we conjugated 20 nm NPs with Ova and immobilized conjugated NPs, NPs alone, or Ova onto a nylon membrane. We probed the membrane with anti-Ova antibodies to confirm co-localization of NPs with Ova. Blot dots in which Ova or NPs alone were spotted produced a signal only in the green or the red channels respectively (Figure S5 A, top and middle rows). Dots in which NP-Ova were spotted produced signal in both the green and the red channels and the two colors co-localized perfectly (Figure S5 A, bottom row). Conjugating 20 nm NPs to Ova did not inhibit their uptake (Figure S5 B, C), moreover Ova and NPs were detected co-localizing 30 minutes after administration in the lumen of the SI and in circulation of the SI on the serosal side (Figure S5 D-F, (white arrows)).

## Discussion

Several routes of antigen uptake from the intestinal lumen have been reported and it is becoming apparent that the mode of antigen uptake plays a role in ensuing immune responses. Whether IECs (enterocytes) play a role in sampling lumen antigens in vivo is not known, although cultured IECs can internalize bacteria, flagellin, peptidoglycan, LPS, and particles of various sizes [27], [28]. Here we have used in vivo imaging of the SI by two-photon/confocal microscopy and IFM of cryosections to examine whether IECs internalize particulate lumen antigens such as viruses, bacterial cell debris, and dietary particles. Initially we used fluorescent *E. coli* as a model particulate antigen and did not observe any appreciable uptake by cells of the epithelium or a significant accumulation of *E.coli* in the LP of the villi. However, *E.coli* cell debris produced by sonication did accumulate in the LP of the villi, but it did not appear to enter via GAPs, indicating that the size of particulate antigen was important for its internalization via GAPs. Sonication of *E. coli* produces cell debris consisting of particulate antigens of varying sizes, making it impossible to examine whether and how the particle size affects their internalization in vivo. Thus, for further studies we used fluorescent particles of known sizes which do not exhibit any endogenous binding affinity to IECs. We found that the uptake of NPs in the SI and their transport was rapid: within 30 minutes of administration to the SI, NPs could be observed in the LP, within the serosal circulation, and in the lymphatics of the SI. In the LP of the villi, NPs co-localized with CD11c+ DCs, although in cryosections of the SI we did not observe any instance of a direct uptake of lumen NPs by CD11c+ DCs of the villi. More recently it was reported that LP CD103+ CD11c+ DCs take up bacterial antigens from the lumen of the SI. We consistently observed NPs located on the basolateral side of the IECs in vivo and using IFM found that a very small number of CD103+ DCs populate the LP of the villi in a steady-state, leading us to consider that IECs could play a role in NP internalization. Although we do find NPs co-localizing with CD11c+ DCs in the LP, we cannot exclude the possibility that some NPs may enter the interstitial spaces, then the blood or lymphatic ducts of the villi, enabling their rapid transport to the MLNs. We found that in addition to Ova and dextran, *Salmonella* LPS enters the LP mainly via GAPs, indicating that in steady-state GAPs may play a role in internalization of small microbial antigens originating from microflora. In vivo imaging of IECs highlighted by NP fluorescence revealed a network of conduit-like structures on the surface of the villi and Peyer's patches that appear to guide soluble antigens into the GAPs. These structures become apparent when highlighted by fluorescent soluble antigens for in vivo imaging, but are not readily discernible in cryosections of the SI. Conduit-like structures do not represent paracellular leakage of the antigen, since they are on the surface of the villi and almost always reach the LP via GAPs. Paracellular leakage of antigen is observed in deceased animals, when the tight junctions lose integrity and the antigen leaks into the LP. In that scenario leakage of fluorescein-labeled dextran appears as fine green lines of fluorescence between all IECs, which is not observed when the SI is imaged in a living animal. Since conduit-like structures are found almost always connected to GAPs, we speculate that they may play a role in directing small soluble antigen to GAPs or alternatively, they might serve to transport/distribute mucus on the surface of the epithelium. The internalization of NPs was most pronounced in the upper 3^rd^ of the villi, especially at the tips of the villi which appear as red patches due to the presence of NPs. The presence of mucus in the intestines makes it difficult for dietary particles, resident microflora, and their products to contact the surface of the IEC. We found that 20 and 40 nm NPs cross the mucus layer and make direct contact with IECs. Our finding is in agreement with reports that capsid virus-like particles such as Norvalk virus (20–30 nm) and human papilloma virus (∼55 nm) diffuse through the cervical mucus as rapidly as in water, while larger (180 nm) herpes simplex virus diffuses 100–1000 times slower in the mucus compared to water [20]. In addition to being covered by mucus, on their apical side IECs have microvilli which could further hinder their contact with larger particles. We observed 40 nm NPs in the endocytic domains between the IEC microvilli, which suggested that small NPs could be internalized by an active endocytic mechanism. In cultured cells the particle size was shown to determine the extent and the pathway of their uptake. Latex particles <200 nm are taken up via clathrin-mediated endocytosis and this uptake can be inhibited by CPZ [28], [29]. As reported for cultured epithelial cells [28], we found that the internalization of 40 nm NPs by IECs can be inhibited by CPZ in vivo. However, to markedly inhibit the NP uptake at least a 5-fold higher concentration of CPZ was necessary. CPZ inhibits clathrin-mediated endocytosis [30], but can also interact with intracellular lipids and cytoskeletal regulators, thus it may affect the uptake of substrates via fluid-phase macropinocytosis [31]. The use of endocytosis inhibitors is still a preferred tool for in vivo studies. However the potentially poor specificity of some inhibitors should always be considered when results of in vivo studies are interpreted. Although CPZ is not an absolutely specific inhibitor of clathrin-mediated-endocytosis [30], the use of CPZ is a good initial approach for distinguishing clathrin-mediated from other endocytic pathways. In several experiments we observed that the amount of 40 nm NPs in the MLNs was significantly decreased (but not abolished) by CPZ, while the entry of dextran via GAPs was unaffected. The uptake of NPs by M cells in the Peyer's patches may account for the presence of NPs in the MLNs of CPZ-treated mice however, we do not have experimental data on whether CPZ affects antigen uptake by M cells. In addition, the varying presence of chyme and the mixing of the intestinal contents in vivo due to intestinal peristalsis may lead to an uneven distribution and absorption of the small volume of PBS/CPZ and NPs in various areas of the SI. Therefore, delineating the endocytic pathways by which IECs internalize NPs in vivo still remains a daunting task. Under similar experimental conditions, internalization of 20 nm NPs was unaffected by administration of CPZ or genistein, even when used at higher concentrations. These findings suggest that depending on their size, NPs may be internalized via distinct pathways, as reported for cultured cells [28]. This possibility is supported by the observation that Ova antigen conjugated to 20 nm NPs reaches the LP and the serosa of the SI in an immunologically relevant form and can be detected with anti-Ova antibodies. We do not believe that administration of scopolamine for in vivo imaging has a significant effect on NP internalization for two reasons. First, scopolamine was administered 10–15 minutes before imaging and at least 20–30 minutes after NP administration and second, no scopolamine was administered to mice that were used for quantifying the NPs in the MLNs by IFM and by spectrofluorophotometry.

NPs and virus-like particles are increasingly being used as vehicles for delivery of antigens and targeted delivery of drugs [32], [33]. Larger sized NPs (>200 nm) have been studied extensively due to their ability to carry larger amounts of cargo, however, there has been little work done in using NPs smaller than 100 nm for these applications. Further characterizing the uptake and trafficking of the NPs and the immune responses to NP-conjugated antigens will be important for understanding how tolerance and immunity to intestinal antigens are generated. This work will also be important for the development of more effective mucosal vaccines and therapies.

## Supporting Information

Figure S1 **A Z-stack image of conduit-like structures on the surface of Peyer's patches highlighted by (A) dextran-fluorescein (green) and (B) dextran-fluorescein (green) and 20 nm NPs (red).** Images are representative of at least 3 experiments.
(TIF)

Figure S2 **Localization of NPs between the microvilli of the IECs imaged with TEM.** NPs were administered into the SI and 40 minutes later the SI was excised and processed for imaging with TEM. (A, B) TEM images of 40 nm NPs lodged between the microvilli (white arrows) of the IECs (40,000X).
(TIF)

Figure S3 **The uptake and distribution of NPs of various sizes in the SI.** (A) The uptake of 20 nm NPs (red) in Peyer's patches 30 minutes after administration in the SI. (B) The uptake of 100 nm NPs (red) in the Peyer's patches 30 minutes after administration in the SI. The follicle-associated epithelium is shown within white rectangles; location of the lumen is denoted with an asterisk. (C) Serosal location of 20 nm NPs (red) and 40 nm NPs (green) 30 minutes after per-oral administration (inset: higher magnification). (D) Serosal location of 40 nm NPs (green) and 1000 nm NPs (red) 30 minutes after administration in the SI. Large amount of 40 nm NPs are seen in serosa (green), but very few 1000 nm NPs (white arrows). (E) A representative image of large NPs (500 nm, red) clumped in mucus. (A–E) Tissue architecture was highlighted by staining with actin-binding phalloidin-Alexa 350 (blue). Lumen of the SI in A, B and E is denoted with asterisks. Images are representative of 3 experiments.
(TIF)

Figure S4 **The uptake of 40, 100, and 500 nm NPs in mouse SI examined by confocal microscopy in vivo.** (A) Thirty Z stack images of the villi were overlayed showing distribution of dextran (green) in the lumen of the SI (asterisk) and 40 nm NPs (red) in the LP. Nuclei of the IECs stained with DAPI (blue). Inset: A higher resolution Z-stack of a single villus showing localization of 40 nm NPs in close proximity to the IEC nuclei (circled); (B) Distribution of 100 nm NPs (red) in the lumen of SI 1 hour after per-oral administration. (C) Distribution of 500 nm NPs (red) in the lumen of SI 1 hour after per-oral administration. (D, E) Higher magnification images of villi from panels B (100 nm NPs) and C (500 nm NPs). Large NPs (100 and 500 nm) are localized in the lumen (asterisks) and do not enter the LP of the villi. Images are representative of 3 experiments.
(TIF)

Figure S5 **The uptake of Ova-conjugated fluorescent NPs (NP-Ova) in the SI 40 minutes after administration in the lumen.** (A) Ova, 20 nm NPs, and 20 nm NP-Ova were spotted on a nylon membrane then probed with rabbit anti-Ova primary and goat anti-rabbit-FITC secondary antibodies (green). The membrane was imaged with a fluorescent microscope at 2.5×. Top row: Ova protein (green); Middle row: 20 nm NPs (red); Bottom row: 20 nm NP-Ova (green and red). 1st column: green channel (FITC); 2nd column-red channel (PE); 3rd column-overlap. (B, C) Internalization of 20 nm NP-Ova in a villus of SI imaged in vivo 40 minutes after intraluminal administration of NP-Ova. Dextran highlights the lumen (B, C (asterisks)), while NPs are found in the SI lumen and the LP (white arrow) of the villi (C). (D–F) An IFM image showing the location of Ova (D, green) and NPs (E, red) in the lumen (asterisks) and serosa (white arrow) of SI tissue sections 40 minutes after NP-Ova administration. (F) Overlap of panels D and E showing co-localization of 20 nm NPs with Ova. Cryosections of the SI were stained with rabbit anti-Ova primary and goat anti-rabbit-FITC secondary antibodies (green). Significant proportion of 20 nm NPs (red) co-localize with Ova (green) in the lumen (asterisk) and serosa (arrow) of the SI. Images are representative of at least 3 experiments.
(TIF)

Movie S1 **Location of 40 nm NPs in a SI villus 30 minutes after administration.** 3-D rendering of Z-stacks showing the location of NPs in the lumen and within the LP of a villus imaged with a confocal microscope.
(ZIP)

Movie S2 **Location of 40 nm NPs and DAPI in the villi of the SI 30 minutes after administration into the lumen.** Dextran was injected i.v. via a tail vein. Z-stacks of images taken from the tip of the villi to approximately 80 µm depth in each fluorescent channel are played sequentially. Blue channel: DAPI-stained nuclei of IECs; Green channel: Dextran-fluorescein; Red channel: 40 nm NPs.
(ZIP)

Movie S3 **Entry of dextran into the LP of the villi via GAPs.** Dextran and 20 nm NPs were administered into the SI lumen and 40 minutes later the lumen side of the intestine was imaged in vivo with a confocal microscope. Dextran (green) can be seen entering the LP via GAPs, while NPs highlight the IECs and can be seen in the LP of the villi. Movie shows 27 Z stacks taken from the tip of the villi to a depth of 70 µm.
(ZIP)

Movie S4 **Conduit-like structures on the villi and entry of dextran into the LP of the villi via GAPs.** 3-D rendering of 27 Z-stacks showing the distribution of GAPs highlighted by dextran (green) and IECs highlighted by 20 nm NPs (red).
(ZIP)

Movie S5 **3-D rendering of Z-stack images taken 40 minutes after 40 nm NP administration in the SI in vivo.** DAPI (blue) enters the IECs and stains the nuclei. 40 nm NPs are internalized and are seen in the LP co-localizing with blood and lymphatic vessels in the villi.
(ZIP)
