# Supplementary figures and images for: The Uptake of Soluble and Particulate Antigens by Epithelial Cells in the Mouse Small Intestine
Source: PLoS One. 2014 Jan 27;9(1):e86656. doi: 10.1371/journal.pone.0086656 (PMC3903549; doi:10.1371/journal.pone.0086656)

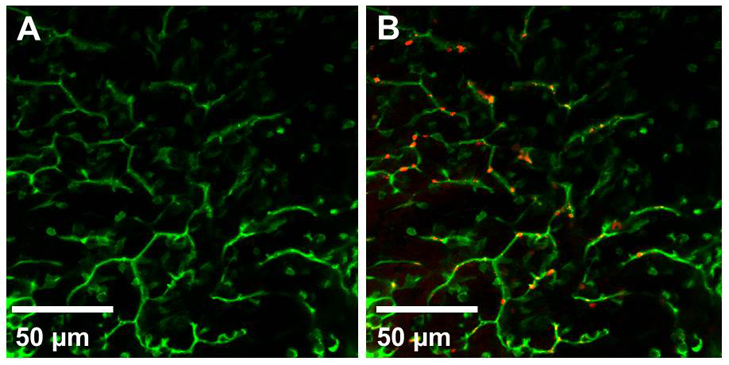

Supplement: Figure S1 — A Z-stack image of conduit-like structures on the surface of Peyer's patches highlighted by (A) dextran-fluorescein (green) and (B) dextran-fluorescein (green) and 20 nm NPs (red). Images are representative of at least 3 experiments. (TIF) [file pone.0086656.s001.tif]

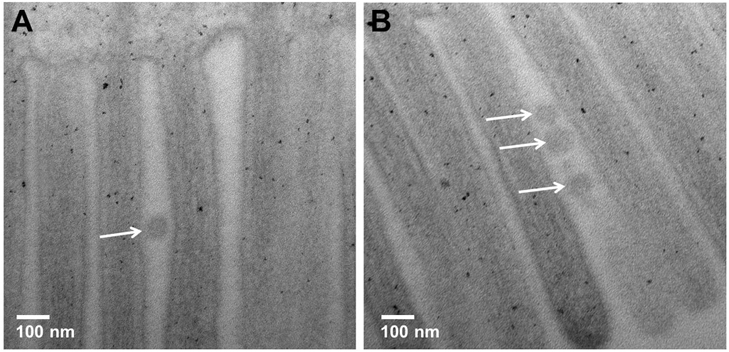

Supplement: Figure S2 — Localization of NPs between the microvilli of the IECs imaged with TEM. NPs were administered into the SI and 40 minutes later the SI was excised and processed for imaging with TEM. (A, B) TEM images of 40 nm NPs lodged between the microvilli (white arrows) of the IECs (40,000X). (TIF) [file pone.0086656.s002.tif]

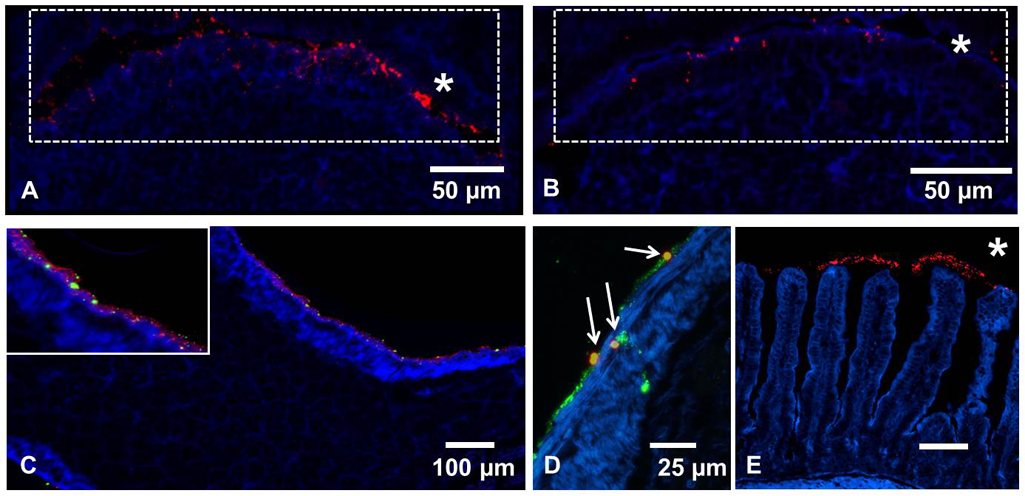

Supplement: Figure S3 — The uptake and distribution of NPs of various sizes in the SI. (A) The uptake of 20 nm NPs (red) in Peyer's patches 30 minutes after administration in the SI. (B) The uptake of 100 nm NPs (red) in the Peyer's patches 30 minutes after administration in the SI. The follicle-associated epithelium is shown within white rectangles; location of the lumen is denoted with an asterisk. (C) Serosal location of 20 nm NPs (red) and 40 nm NPs (green) 30 minutes after per-oral administration (inset: higher magnification). (D) Serosal location of 40 nm NPs (green) and 1000 nm NPs (red) 30 minutes after administration in the SI. Large amount of 40 nm NPs are seen in serosa (green), but very few 1000 nm NPs (white arrows). (E) A representative image of large NPs (500 nm, red) clumped in mucus. (A–E) Tissue architecture was highlighted by staining with actin-binding phalloidin-Alexa 350 (blue). Lumen of the SI in A, B and E is denoted with asterisks. Images are representative of 3 experiments. (TIF) [file pone.0086656.s003.tif]

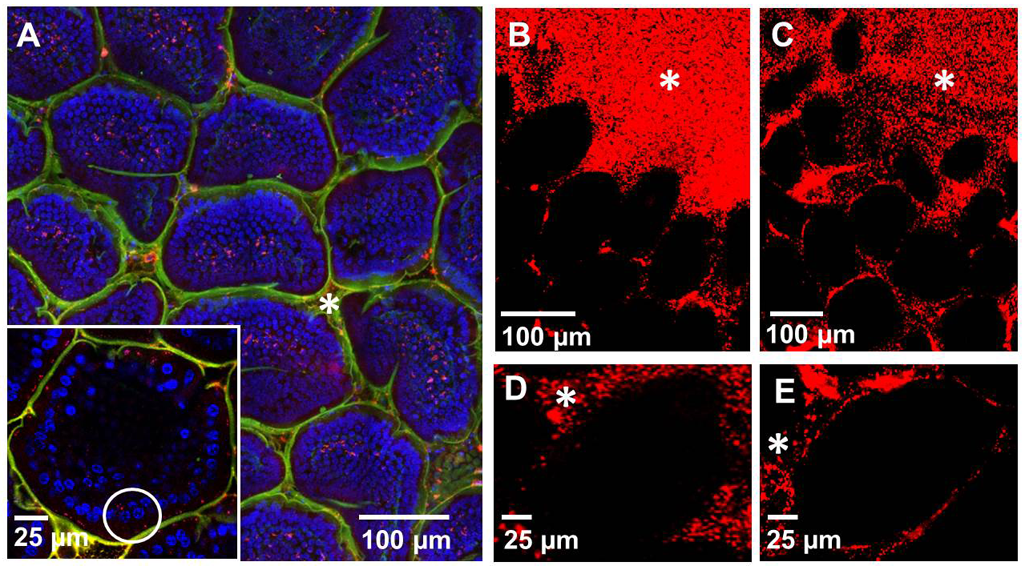

Supplement: Figure S4 — The uptake of 40, 100, and 500 nm NPs in mouse SI examined by confocal microscopy in vivo. (A) Thirty Z stack images of the villi were overlayed showing distribution of dextran (green) in the lumen of the SI (asterisk) and 40 nm NPs (red) in the LP. Nuclei of the IECs stained with DAPI (blue). Inset: A higher resolution Z-stack of a single villus showing localization of 40 nm NPs in close proximity to the IEC nuclei (circled); (B) Distribution of 100 nm NPs (red) in the lumen of SI 1 hour after per-oral administration. (C) Distribution of 500 nm NPs (red) in the lumen of SI 1 hour after per-oral administration. (D, E) Higher magnification images of villi from panels B (100 nm NPs) and C (500 nm NPs). Large NPs (100 and 500 nm) are localized in the lumen (asterisks) and do not enter the LP of the villi. Images are representative of 3 experiments. (TIF) [file pone.0086656.s004.tif]

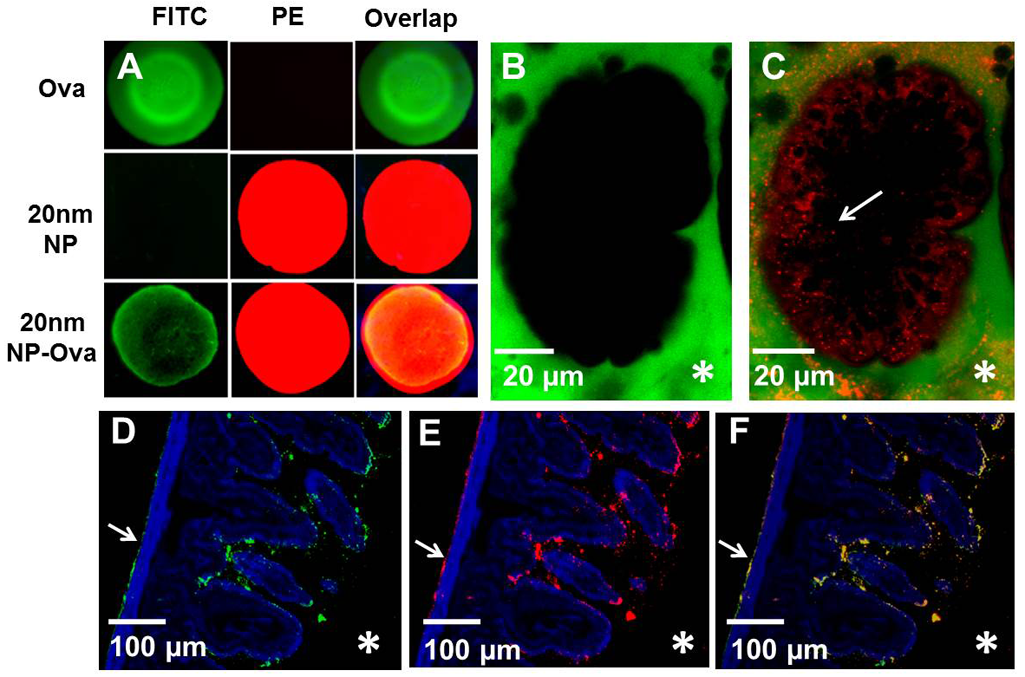

Supplement: Figure S5 — The uptake of Ova-conjugated fluorescent NPs (NP-Ova) in the SI 40 minutes after administration in the lumen. (A) Ova, 20 nm NPs, and 20 nm NP-Ova were spotted on a nylon membrane then probed with rabbit anti-Ova primary and goat anti-rabbit-FITC secondary antibodies (green). The membrane was imaged with a fluorescent microscope at 2.5×. Top row: Ova protein (green); Middle row: 20 nm NPs (red); Bottom row: 20 nm NP-Ova (green and red). 1st column: green channel (FITC); 2nd column-red channel (PE); 3rd column-overlap. (B, C) Internalization of 20 nm NP-Ova in a villus of SI imaged in vivo 40 minutes after intraluminal administration of NP-Ova. Dextran highlights the lumen (B, C (asterisks)), while NPs are found in the SI lumen and the LP (white arrow) of the villi (C). (D–F) An IFM image showing the location of Ova (D, green) and NPs (E, red) in the lumen (asterisks) and serosa (white arrow) of SI tissue sections 40 minutes after NP-Ova administration. (F) Overlap of panels D and E showing co-localization of 20 nm NPs with Ova. Cryosections of the SI were stained with rabbit anti-Ova primary and goat anti-rabbit-FITC secondary antibodies (green). Significant proportion of 20 nm NPs (red) co-localize with Ova (green) in the lumen (asterisk) and serosa (arrow) of the SI. Images are representative of at least 3 experiments. (TIF) [file pone.0086656.s005.tif]
